# Driving Cerebellar Theta Oscillations Interferes With Voluntary Neck Movements in Cervical Dystonia

**DOI:** 10.1002/mds.70394

**Published:** 2026-06-10

**Authors:** Davide Costa, Andrea Guerra, Daniele Birreci, Massimiliano Passaretti, Martina De Riggi, Anna Sofia Grandolfo, Valentina D'Onofrio, Luca Angelini, Giulia Paparella, Matteo Bologna

**Affiliations:** ^1^ Department of Human Neurosciences Sapienza, University of Rome Rome Italy; ^2^ Department of Neuroscience University of Padua Padua Italy; ^3^ Padova Neuroscience Center University of Padua Padua Italy; ^4^ Department of Clinical Neuroscience Karolinska Institutet Stockholm Sweden; ^5^ IRCCS Neuromed Pozzilli Italy; ^6^ Neurophysiopathology Unit, Headache Center, DiBrain Department Bari Aldo Moro University, Policlinico General Hospital Bari Italy

**Keywords:** Cerebellum, Dystonia, Neuromodulation, Oscillations, Theta

## Abstract

**Background:**

Cervical dystonia (CD) is a movement disorder with a complex pathophysiology, including cerebellar abnormalities. Transcranial alternating current stimulation (tACS), a noninvasive neuromodulation technique capable of entraining brain oscillations, can transiently modulate neuronal activity and enhance resonant rhythms.

**Objective:**

The aim of this study was to explore whether cerebellar tACS delivered at specific cerebellar‐resonant frequencies modifies fast voluntary neck movements in patients with CD and whether botulinum toxin (BoNT) therapy influences tACS effects.

**Methods:**

Eighteen patients with CD, predominantly exhibiting the torticollis phenotype, were included. Fast voluntary neck movements were objectively assessed using motion analysis during two experimental sessions: (1) pre‐BoNT and (2) 1 month after BoNT. Cerebellar tACS was applied at theta (θ), beta (β), and gamma (γ) frequencies, along with sham stimulation, while patients performed fast neck movements. Peak angular velocity and angular amplitude of both prodystonic and antidystonic movements were measured.

**Results:**

In the OFF‐BoNT state, neck (antidystonic) movements' velocity and amplitude decreased with θ‐tACS, particularly when stimulation was applied to the cerebellar hemisphere ipsilateral to the side of torticollis. BoNT ameliorated movement velocity and amplitude, but it did not change the detrimental effect of cerebellar θ oscillations on antidystonic movements.

**Conclusions:**

Driving cerebellar θ oscillations interferes with the execution of fast voluntary neck movements in CD, and BoNT therapy does not influence this effect. These findings support the view of dystonia as a network disorder in which the cerebellum plays a key role. © 2026 The Author(s). *Movement Disorders* published by Wiley Periodicals LLC on behalf of International Parkinson and Movement Disorder Society.

Dystonia is a movement disorder characterized by sustained or intermittent abnormal movements, postures, or both.^1^ Cervical dystonia (CD) represents one of the most common forms of adult‐onset dystonia in clinical practice.^2^, ^3^ CD not only compromises patients' quality of life by causing pain and functional limitations but also presents significant challenges in therapeutic management, particularly in cases refractory to conventional treatments.^4^

The pathophysiology of CD is complex and not yet fully understood.^5^, ^6^, ^7^ Historically, CD was primarily considered a basal ganglia disorder, with pathophysiological models emphasizing abnormal activity within pallidal circuits and their therapeutic modulation through lesioning or deep brain stimulation.^8^ Growing evidence suggests a significant involvement of the cerebellum in CD pathogenesis,^9^, ^10^, ^11^ supported by anatomical and functional connections between cerebellar and basal ganglia circuits.^12^ In particular, cerebellar outputs via thalamic nuclei may influence both cortical and striatal activity, contributing to abnormal motor control.^13^ These findings have led to the development of a network‐based view of dystonia, arising from complex and interconnected dysfunctions across multiple brain regions.^14^ Reduced activity in this pathway may contribute to the development of abnormal movements such as CD, providing a rationale for cerebellar transcranial Direct Current Stimulation (tDCS) as a potential therapeutic approach.^15^, ^16^ Neurophysiological studies in dystonia have reported abnormal oscillatory activity, which is thought to reflect altered functional interactions within these distributed networks.^17^, ^18^ Local field potential recordings in patients with dystonia who are undergoing deep brain stimulation show abnormal basal ganglia–thalamic activity, including altered firing patterns and elevated resting pallidal theta (θ) power.^18^, ^19^ Globus pallidus internus–cortical coherence confirmed prominent low‐frequency activity at rest in patients with dystonia,^20^ supporting the hypothesis that enhanced low‐frequency oscillations may contribute to the generation of abnormal movements.^21^

Transcranial alternating current stimulation (tACS) is a noninvasive technique that entrains brain oscillations and enhances their power when applied at the area's endogenous rhythm.^22^, ^23^, ^24^ tACS offers a unique setting to investigate the contribution of specific rhythmic activity in the brain cortex and in the cerebellum of patients with CD.^25^, ^26^, ^27^ The cerebellum displays a range of oscillatory activities, from low‐frequency θ and beta (β) rhythms in granule cells to higher‐frequency gamma (γ) oscillations linked to Purkinje cell activity.^28^, ^29^, ^30^ However, despite these intrinsic rhythmic patterns representing potential targets for neuromodulatory interventions, the effects of cerebellar tACS in CD remain unexplored.

In this study, we explored the effects of cerebellar tACS in patients with CD, aiming to probe the contribution of cerebellar oscillatory activity to dystonia pathophysiology and to evaluate its effects on motor control, along with its interaction with botulinum toxin (BoNT) treatment. We tested different stimulation frequencies hypothesized to resonate with intrinsic cerebellar rhythms, allowing us to explore the functional role of multiple oscillatory activities. Both prodystonic and antidystonic movements were examined to dynamically assess how cerebellar neuromodulation influences motor control. tACS was delivered ipsilateral and contralateral to the clinically affected side, enabling exploration of potential hemispheric asymmetries. Patients were studied before and after BoNT treatment, allowing evaluation of the disease state and possible interactions with the current gold standard therapy. Overall, the study addresses key gaps in understanding cerebellar contributions to dystonia from a pathophysiological perspective.

## Subjects and Methods

### Participants

We recruited 18 patients with CD (12 females; mean age ± standard deviation [SD]: 62.1 ± 12.9 years) and 12 age‐ and sex‐matched healthy control subjects (HCs; 6 females; mean age ± SD: 57.8 ± 15.5 years). Patients were consecutively enrolled at the Department of Human Neurosciences, Sapienza University of Rome, and their diagnosis was established according to current clinical criteria.^31^ Clinical evaluation included administration of the Toronto Western Spasmodic Torticollis Rating Scale (TWSTRS)^32^ (further details are reported in the Supporting Information Materials and Methods). Experimental procedures were conducted in accordance with the Declaration of Helsinki, following international safety guidelines,^33^ and were approved by the local ethics committee. Written informed consent was obtained from all participants before their inclusion in the study.

### tACS

tACS was delivered using a BrainSTIM device (E.M.S.) connected to two 5 × 5 cm electrodes embedded in saline‐soaked sponges, at a stimulation frequency of 5 Hz for θ, 20 Hz for β, and 50 Hz for γ tACS. To ensure that the electric field was predominantly confined to the targeted cerebellar region, we based the stimulation parameters on previous studies in which realistic head‐model simulations were performed.^27^ Specifically, one electrode was positioned at the center of the cerebellar hemisphere (~1 cm below and 3 cm lateral to the inion) and the other electrode over the ipsilateral buccinator muscle^25^, ^26^ (further details are provided in the Supporting Information and Methods).

### Kinematic Recordings

We used an optoelectronic system to track six reflective markers placed on the trunk and head. Participants were asked to perform rotational neck movements “as fast and wide as possible” (further details are provided in Supporting Information Materials and Methods). Peak angular velocity (degree/s) and movement amplitude (degrees) were measured.^34^ For each parameter, variability was quantified using the coefficient of variation (CV). In addition, to assess within‐subject temporal variability, we calculated the CV of the mean intermovement interval (i.e., the time between consecutive head movements).

### Experimental Design

The study consisted of two randomized, counterbalanced experimental sessions, including clinical and kinematic assessments (Fig. 1). One session occurred at least 3 months after BoNT injection (pre‐BoNT) and the other during maximal BoNT efficacy, approximately 1 month post‐injection (post‐BoNT). In each session, the motor task was recorded under four conditions: θ‐, β‐, γ‐, and sham‐tACS. Fast neck movements comprised one trial of five prodystonic and one trial of five antidystonic rotations per stimulation condition and cerebellar hemisphere (ipsilateral and contralateral to the torticollis side), totaling 16 trials per session. To collect normative kinematic values of head movements, we also enrolled a group of HCs, and they took part in one experimental session consisting of five head movements to the left and five to the right during sham‐tACS (further details are provided in the Supporting Information Materials and Methods).

**FIG. 1:**
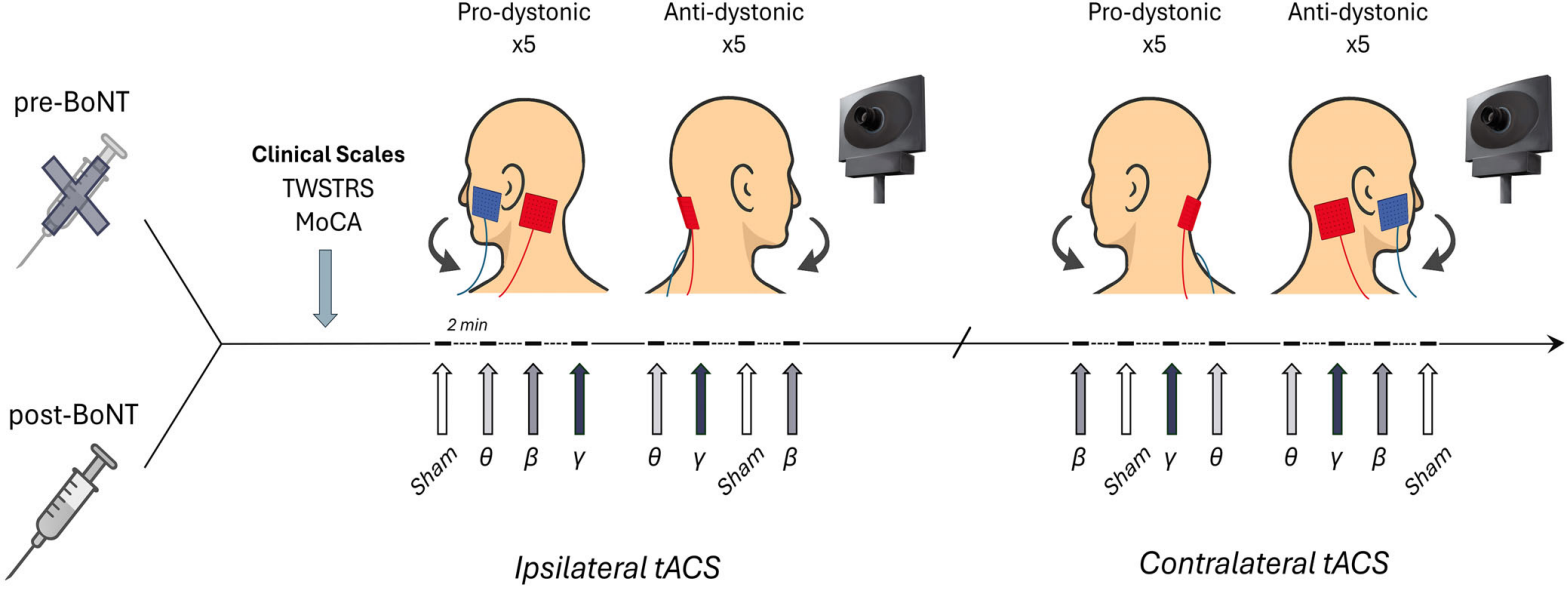
Experimental protocol. Participants completed two experimental sessions: one session ≥3 months after botulinum toxin injection (pre‐BoNT) and one session ≈1 month after treatment (post‐BoNT). Each session included clinical (Toronto Western Spasmodic Torticollis Rating Scale [TWSTRS], Montreal Cognitive Assessment [MoCA]) and kinematic assessments during θ‐, β‐, γ‐, and sham‐tACS (transcranial alternating current stimulation) applied over the ipsilateral and contralateral cerebellar hemispheres relative to the torticollis side (leftward in the figure). For each stimulation condition, participants performed five prodystonic (toward torticollis) and five antidystonic (opposite) head rotations. Each trial was followed by a rest period (2 minutes) to prevent fatigue. Stimulation order was randomized and double blind for participants and investigators. [Color figure can be viewed at wileyonlinelibrary.com]

### Statistical Analysis

Mann–Whitney *U* tests were used to compare age and clinical scores between patients with CD and HCs, while Wilcoxon tests assessed TWSTRS score changes pre‐ and post‐BoNT. Cochran's *Q* test examined differences in the proportion of patients reporting side effects across stimulation conditions. Kinematic parameters during sham‐tACS were compared between patients and HCs using unpaired *t* tests, with patient values averaged across ipsilateral and contralateral stimulation. In HCs, given that movements to the left and right sides are expected to be performed at similar speed and amplitude in the absence of pathological conditions,^34^ we used the average of both sides for statistical analysis. The effects of tACS on kinematic parameters in patients with CD pre‐BoNT were analyzed using repeated‐measures analyses of variance (ANOVAs; rmANOVA) with the within‐subject factors “stimulation side” (ipsilateral, contralateral), “stimulation frequency” (θ‐, β‐, γ‐, sham‐tACS), and “movement direction” (prodystonic, antidystonic). As a control, we replaced “stimulation side” with “stimulated hemisphere” (right, left) to test whether effects depended solely on the cerebellar hemisphere. To assess BoNT influence, an rmANOVA included the factors “session” (pre‐BoNT, post‐BoNT), “stimulation frequency,” and “movement direction.” Post hoc comparisons were performed using Duncan's multiple range test. For rmANOVAs, sphericity was assessed using Mauchly's test, and Greenhouse–Geisser corrections were applied when the assumption was violated. Spearman correlations evaluated relationships between kinematic measures and clinical scores, with tACS effects quantified as the ratio of real to sham stimulation values. Percentage changes in TWSTRS scores and tACS effects between sessions were computed as [(post–pre)/pre × 100]. Statistical significance was set at *P* < 0.05. Values are reported as mean ± SD unless otherwise specified. Analyses were conducted using Statistica (TIBCO Software).

## Results

Of the 18 patients enrolled, 16 completed both experimental sessions, while 2 completed only the pre‐BoNT session for personal reasons, unrelated to the experimental procedures or stimulation, which prevented completion of the study protocol within the required timeframe. Cutaneous sensations were reported by three subjects during sham‐tACS, six during γ‐tACS, five during θ‐tACS, and five during β‐tACS, with no significant differences across conditions (*Q* = 3.00, *P* = 0.39); all were mild (score ≤ 3) and caused no discomfort. No visual sensations occurred during sham or θ‐tACS, and only 1 subject reported them during γ‐tACS. Five patients reported mild visual sensations during β‐tACS (*Q* = 12.75, *P* < 0.01), which did not interfere with the task. There were no significant differences between patients and HCs in age (*P* = 0.29), sex distribution (*P* = 0.38), or Montreal Cognitive Assessment scores (*P* = 0.39). As expected, TWSTRS scores were higher pre‐BoNT than post‐BoNT (30.7 ± 17.5 vs. 19.8 ± 13.1; *P* < 0.0001).

### Effects of Cerebellar‐tACS on Neck Movements

As expected, patients with CD (pre‐BoNT) performed slower and fewer wide neck movements compared with HCs, both when considering antidystonic (velocity HC: 428.9 ± 70.8, CD: 245.7 ± 118.9 degrees/s; *P* < 0.001; amplitude HC: 57.2 ± 11.5 degrees, CD: 38.8 ± 16.1 degrees; *P* = 0.001) and prodystonic movement directions (velocity CD: 232.4 ± 97.0 degrees/s, *P* < 0.001; amplitude CD: 45.2 ± 15.0 degrees, *P* = 0.019) (Table 1).

**TABLE 1:** Neck rotational movements in patients with cervical dystonia before (pre‐BoNT) and after (post‐BoNT) therapy with botulinum toxin type A during sham‐tACS

	Patients			
	Prodystonic		Antidystonic	
	Pre‐BoNT	Post‐BoNT	Pre‐BoNT	Post‐BoNT
Angular amplitude (degrees)	46.4 ± 15.3	43.9 ± 14.4	38.9 ± 16.5	44.2 ± 19.1*
Peak angular velocity (degree/s)	240.7 ± 99.1	266.6 ± 110.2*	252.1 ± 124.4	283.2 ± 128.3*

*Note*: Values refer only to the 16 patients who completed both experimental sessions. Values are expressed as mean ± 1 standard deviation.

Abbreviations: BoNT, botulinum toxin; tACS, transcranial alternating current stimulation.

* *P* < 0.05 vs. pre‐BoNT.

In the pre‐BoNT state, neck movement velocity and amplitude were modulated by tACS depending on stimulation frequency, side, and movement direction, as shown by a significant “stimulation frequency” × “stimulation side” × “movement direction” interaction (velocity: *F*_3,51_ = 2.88, *P* = 0.045, *η* ^2^ = 0.145; amplitude: *F*_3,51_ = 4.02, *P* = 0.012, *η* ^2^ = 0.191). Post hoc analyses demonstrated that θ‐tACS over the cerebellar hemisphere ipsilateral to the torticollis direction reduced the velocity and amplitude of antidystonic movements compared with sham (velocity: *P* = 0.010; amplitude: *P* = 0.005), β‐tACS (velocity: *P* = 0.032; amplitude: *P* = 0.033), and γ‐tACS (amplitude: *P* = 0.013; velocity: *P* = 0.112) (Fig. 2). No changes in kinematic parameters were observed across stimulation frequencies for prodystonic movements or when tACS was applied over the cerebellar hemisphere contralateral to the torticollis direction. As expected, rmANOVA showed that movement amplitude was generally lower for antidystonic than prodystonic movements (main effect of “movement direction”: *F*_1,17_ = 5.08, *P* = 0.038, *η* ^2^ = 0.230), with no significant effects of other main factors or interactions (Table 2). Additional rmANOVAs replacing “stimulation side” with “stimulated hemisphere” showed no “stimulation frequency” × “stimulated hemisphere” (velocity: *F*_3,51_ = 0.25, *P* = 0.862; amplitude: *F*_3,51_ = 2.02, *P* = 0.122) or “stimulation frequency” × “stimulated hemisphere” × “movement direction” interactions (velocity: *F*_3,51_ = 0.60, *P* = 0.615; amplitude: *F*_3,51_ = 0.37, *P* = 0.775), indicating that stimulating a specific cerebellar hemisphere is irrelevant unless the individual dystonia pattern (torticollis direction) is considered. In addition, trial‐to‐trial variability, assessed via the CV of peak velocity, movement amplitude, and the intermovement interval, did not differ across stimulation conditions, indicating consistent performance across trials (detailed analyses are provided in the Supporting Information Materials and Methods).

**FIG. 2:**
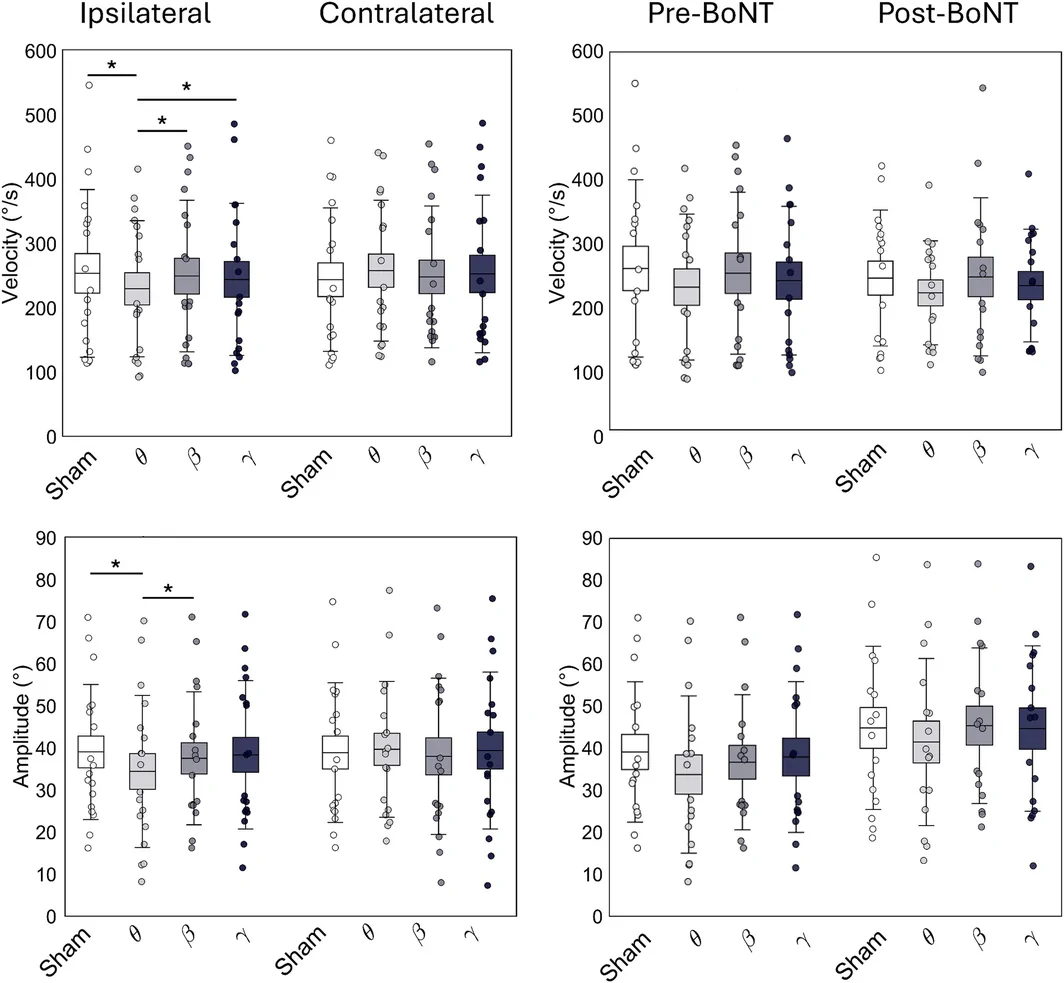
Effects of cerebellar transcranial alternating current stimulation (tACS) on kinematic characteristics of antidystonic neck movements. Box plots show individual and group data for velocity (upper panels) and amplitude (lower panels) of antidystonic movements during sham, θ‐, β‐, and γ‐tACS. Left panels: data from all patients for stimulation of the cerebellar hemisphere ipsilateral and contralateral to the direction of torticollis in the session before botulinum toxin injection (pre‐BoNT). Right panels: data from the 16 patients who completed both pre‐ and post‐BoNT sessions for ipsilateral hemisphere stimulation. Each box represents the mean ± 1 standard error of the mean (SEM), and whiskers indicate the mean ± standard deviation (SD). Asterisks denote statistically significant differences (*P* < 0.05). [Color figure can be viewed at wileyonlinelibrary.com]

**TABLE 2:** Kinematic data and statistics for the neck rotational movements in patients with cervical dystonia before therapy with botulinum toxin type A

Kinematic parameter	Stimulation frequency	Raw data				
		Ipsilateral^a^		Contralateral^b^		
		Prodystonic	Antidystonic	Prodystonic	Antidystonic	rmANOVA
Ang ampl	Sham	44.7 ± 16.5	39.0 ± 16.0	45.6 ± 13.7	38.7 ± 16.6	STIM FREQ: *F*_3,51_: 1.05, *P =* 0.38
	θ	45.0 ± 14.9	34.3 ± 18.1	44.5 ± 14.9	39.5 ± 16.1	STIM SIDE: *F*_1,17_: 1.65, *P =* 0.22
	β	42.8 ± 15.7	37.4 ± 15.8	46.9 ± 14.8	37.8 ± 18.6	MOV DIR: *F*_1,17_: 5.08, ***P =* 0.038**
	γ	45.5 ± 14.9	38.3 ± 17.6	44.8 ± 13.1	39.2 ± 18.6	STIM FREQ × STIM SIDE: *F*_3,51_: 1.04, *P =* 0.38
						STIM FREQ × MOV DIR: *F*_3,51_: 0.36, *P =* 0.79
						STIM SIDE × MOV DIR: *F*_1,17_: 0.19, *P =* 0.67
						STIM FREQ × STIM SIDE × MOV DIR: *F*_3,51_: 4.02, ***P =* 0.012**
Peak vel	Sham	222.4 ± 93.6	253.1 ± 130.3	242.3 ± 102.1	238.4 ± 109.6	STIM FREQ: *F*_3,51_: 0.43, *P =* 0.73
	θ	227.0 ± 95.8	229.2 ± 105.8	242.5 ± 109.1	252.1 ± 107.7	STIM SIDE: *F*_1,17_: 1.28, *P =* 0.27
	β	227.4 ± 101.7	248.9 ± 117.9	241.8 ± 108.6	242.7 ± 108.4	MOV DIR: *F*_1,17_: 0.49, *P =* 0.49
	γ	232.8 ± 97.0	243.6 ± 118.2	242.4 ± 112.9	247.2 ± 120.6	STIM FREQ × STIM SIDE: *F*_3,51_: 2.00, *P =* 0.12
						STIM FREQ × MOV DIR: *F*_3,51_: 0.35, *P =* 0.79
						STIM SIDE × MOV DIR: *F*_1,17_: 1.73, *P =* 0.21
						STIM FREQ × STIM SIDE × MOV DIR: *F*_3,51_: 2.88, ***P =* 0.045**

*Note*: Data reflect mean values ± 1 standard deviation.

Ipsilateral cerebellar hemisphere to the side of dystonia.

Contralateral cerebellar hemisphere to the side of dystonia.

Abbreviations: rmANOVA, repeated‐measures analysis of variance; Ang ampl, angular amplitude (degrees); tACS, transcranial alternating current stimulation; Sham, sham‐tACS; STIM FREQ, factor “stimulation frequency”; θ, theta‐tACS; STIM SIDE, factor “stimulation side”; β, beta‐tACS; MOV DIR, factor “movement direction”; γ, gamma‐tACS; Peak vel, peak of angular velocity (degrees/s). Bold represents significant factors and interactions.

### Interaction Between Cerebellar‐tACS and BoNT Effects on Neck Movements

After BoNT treatment (post‐BoNT), neck movement velocity improved for both antidystonic and prodystonic directions, as indicated by a significant main effect of “session” (*F*_1,15_ = 4.63, *P* = 0.048, *η* ^2^ = 0.236) and no “session” × “movement direction” interaction (*F*_1,15_ = 0.13, *P* = 0.718) (Table 1). Importantly, cerebellar θ‐tACS effects were not influenced by BoNT, with no “session” × “stimulation frequency” (*F*_3,45_ = 0.07, *P* = 0.98) or “session” × “stimulation frequency” × “movement direction” interaction (*F*_3,45_ = 0.82, *P* = 0.49) for ipsilateral stimulation. The “stimulation frequency” × “movement direction” interaction was significant (*F*_3,45_ = 2.94, *P* = 0.043, *η* ^2^ = 0.164), and post hoc tests confirmed that θ‐tACS decreased antidystonic velocity compared with sham (*P* = 0.013) and β‐tACS (*P* = 0.007), with no effect on prodystonic movements (*P* > 0.05) (Fig. 2).

BoNT also improved movement amplitude, with a significant “session” × “movement direction” interaction (F1,15 = 5.14, *P* = 0.039, *η* ^2^ = 0.262). Post hoc tests showed increased amplitude for antidystonic movements (*P* = 0.007), while prodystonic movements were unchanged (*P* = 0.699). Pre‐BoNT, antidystonic movements had lower amplitude than prodystonic ones (*P* = 0.004), but amplitudes were similar post‐BoNT (*P* = 0.930). As with velocity, rmANOVA on amplitude showed no interaction between θ‐tACS and BoNT (“session” × “stimulation frequency”: *F*_3,45_ = 0.90, *P* = 0.448; “session” × “stimulation frequency” × “movement direction”: *F*_3,45_ = 0.08, *P* = 0.972), while confirming detrimental effects of θ‐tACS on antidystonic movements (“stimulation frequency” × “movement direction”: *F*_3,45_ = 6.02, *P* = 0.002, η^2^ = 0.306). Post hoc analysis showed θ‐tACS decreased antidystonic amplitude compared with sham‐tACS (*P* < 0.001), β‐tACS (*P* = 0.003), and γ‐tACS (*P* = 0.002), with no effect on prodystonic movements (*P* > 0.05) (Fig. 2, Table 3). Finally, trial‐to‐trial variability was not affected by BoNT treatment (detailed analyses are provided in the Supporting Information Materials and Methods).

**TABLE 3:** Kinematic data for the neck rotational movements in patients with cervical dystonia after therapy with botulinum toxin type A (BoNT) and statistics related to the effects of cerebellar tACS delivered over the cerebellar hemisphere ipsilateral to the torticollis side (main effect) and BoNT treatment

Kinematic parameter	Stimulation frequency	Raw data		
		Prodystonic	Antidystonic	rmANOVA
Ang ampl	Sham	44.7 ± 15.1	45.4 ± 19.7	STIM FREQ: *F*_3,45_: 1.85, *P =* 0.15
	θ	46.8 ± 13.7	42.0 ± 20.2	SESS: *F*_1,15_: 6.73, ***P =* 0.02**
	β	44.0 ± 16.3	45.9 ± 18.8	MOV DIR: *F*_1,15_: 5.08, ***P =* 0.038**
	γ	46.3 ± 16.3	45.2 ± 20.0	STIM FREQ × MOV DIR: *F*_3,45_: 6.02, ***P =* 0.002**
				STIM FREQ × SESS: *F*_3,45_: 0.90, *P =* 0.448
				SESS × MOV DIR: *F*_1,15_: 5.14, ***P =* 0.039**
				STIM FREQ × SESS × MOV DIR: *F*_3,45_: 0.08, *P =* 0.97
Peak vel	Sham	268.2 ± 111.5	282.0 ± 126.5	STIM FREQ: *F*_3,45_: 1.89, *P =* 0.15
	θ	268.3 ± 99.3	254.7 ± 96.5	SESS: *F*_1,15_: 4.63, ***P =* 0.048**
	β	259.2 ± 121.3	284.2 ± 146.8	MOV DIR: *F*_1,15_: 0.22, *P =* 0.65
	γ	271.9 ± 107.2	268.1 ± 104.6	STIM FREQ × MOV DIR: *F*_3,45_: 2.94, ***P =* 0.043**
				STIM FREQ × SESS: *F*_3,45_: 0.07, *P =* 0.98
				SESS × MOV DIR: *F*_1,15_: 0.13, *P =* 0.72
				STIM FREQ × SESS × MOV DIR: *F*_3,45_: 0.82, *P =* 0.49

*Note*: Data reflect mean values ± 1 standard deviation.

Abbreviations: BoNT, botulinum toxin; tACS, transcranial alternating current stimulation; rmANOVA, repeated‐measures analysis of variance; Ang ampl, angular amplitude (degrees); Sham, sham‐tACS; STIM FREQ, factor “stimulation frequency”; θ, theta‐tACS; SESS, factor “session”; β, beta‐tACS; MOV DIR, factor “movement direction”; γ, gamma‐tACS; Peak vel, peak of angular velocity (degrees/s). Bold represents significant factors and interactions.

### Clinical‐Neurophysiological Correlations

At baseline (pre‐BoNT), TWSTRS severity scores correlated significantly with θ‐tACS effects on movement amplitude (*r* = −0.56, *P* = 0.016), indicating that greater disease severity was associated with a stronger detrimental effect on antidystonic neck movements. A similar, nonsignificant trend was observed for movement velocity (*r* = −0.41, *P* = 0.089) (Fig. 3). No correlations were found between TWSTRS scores and θ‐tACS effects on amplitude (*r* = −0.24, *P* = 0.366) or velocity (*r* = −0.38, *P* = 0.15) post‐BoNT, nor between changes in TWSTRS scores and θ‐tACS effects from pre‐ to post‐BoNT (amplitude: *r* = 0.14, *P* = 0.601; velocity: *r* = −0.12, *P* = 0.651).

**FIG. 3:**
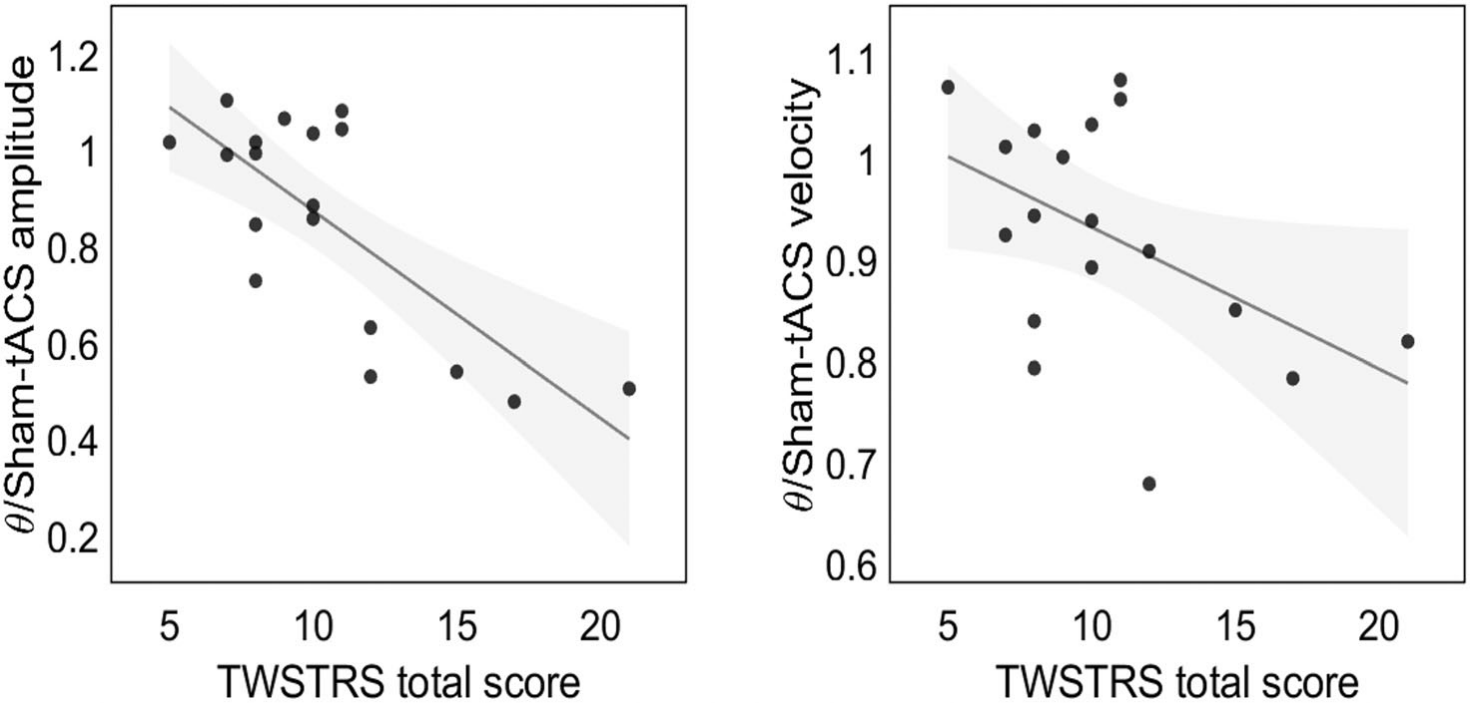
Clinical‐neurophysiological correlations. Spearman's correlations between Toronto Western Spasmodic Torticollis Rating Scale (TWSTRS) total score and the ratio of kinematic parameters of antidystonic movements during θ‐tACS (theta transcranial alternating current stimulation) relative to sham‐tACS in the pre‐BoNT session. A significant negative correlation (*r* = −0.56, *P* = 0.01) was observed between TWSTRS scores and changes in movement amplitude with θ‐tACS (left panel), indicating that the detrimental effects induced by θ‐tACS were stronger in patients with more severe dystonia. A similar relationship was observed for movement velocity (right panel), although it did not reach statistical significance (*r* = −0.41, *P* = 0.08).

## Discussion

In this study, we applied tACS over the cerebellum at cerebellar‐resonant frequencies to modulate rotational neck movements in patients with CD. Patients with CD showed reduced neck movement velocity and amplitude compared with HCs in both antidystonic and prodystonic directions, consistent with previous studies.^34^, ^35^ Several hypotheses have been proposed to explain this alteration, ranging from co‐contraction of agonist and antagonist muscles^5^ to impaired central motor programming.^34^ Neck rotation was smaller in the antidystonic direction, suggesting impaired feedforward and sensory feedback mechanisms that restrict movement range.^36^ In addition, increased effort to suppress dystonic activity may impose an additional motor control load,^37^ further limiting movement range especially in the antidystonic direction. Moreover, we confirm previous literature^38^, ^39^ showing that BoNT treatment improves neck rotation velocity and amplitude in CD, especially in the antidystonic direction.^40^ tACS at θ frequency selectively impaired fast neck movements, especially when applied to the cerebellar hemisphere ipsilateral to the torticollis side and during antidystonic movements. This effect correlated with TWSTRS scores, linking disease severity to cerebellar network vulnerability. BoNT treatment improved neck movements in both antidystonic and prodystonic directions but did not offset the detrimental effects of θ‐tACS.

### Effects of Cerebellar‐tACS on Neck Movements in CD

Remarkably, the observed effects of cerebellar tACS in patients depended on stimulation site, frequency, and movement direction relative to the dystonic pattern. When θ‐tACS was delivered over the cerebellar hemisphere ipsilateral to the torticollis direction, both the velocity and amplitude of antidystonic neck movements decreased (Fig. 4), whereas no effects were observed with prodystonic movements, when the stimulation was delivered over the cerebellar hemisphere contralateral to the torticollis direction, or during β‐tACS or γ‐tACS. tACS synchronizes neuronal oscillations at the stimulation frequency only if it matches the endogenous rhythm of the area being stimulated.^22^, ^23^, ^24^ Previous evidence suggests that cerebellar granule and Golgi cells have natural oscillatory properties in the θ band,^29^, ^41^, ^42^, ^43^, ^44^ and intrinsic cerebellar oscillations play a functional role in cerebrocerebellar communication during sensorimotor processing and movement control.^28^, ^30^ θ oscillations have been consistently implicated in the pathophysiology of dystonia, particularly within the basal ganglia–thalamic circuits, where increased θ power is associated with abnormal motor output and impaired movement control.^18^, ^20^ The cerebellum as a key node in the dystonia network contributes to maladaptive sensorimotor integration and abnormal timing of motor commands.^14^, ^45^ Importantly, altered cerebellar signals in animal models, including abnormal Purkinje cell firing, and dysfunctional interactions with the basal ganglia are involved in generating dystonic movements.^9^, ^46^ Building on these observations, we suggest that pathological oscillatory θ activity is present at the cerebellar level. Cerebellar θ‐tACS may have amplified this pathologically enhanced θ activity within the cerebellar node, leading to further disruption of network dynamics and worsening of head movement in patients with CD (Fig. 4). This finding is consistent with a potential role of θ oscillations in dystonic motor impairments. However, in the absence of direct measurements of neural activity, alternative explanations, such as broader network‐level modulation, cannot be excluded. Also, our findings underscore the cerebellum's active and specific contribution to network‐level dysfunction, in line with a recent probabilistic tractography study assessing cerebellar afferent and efferent pathways in patients with CD who are undergoing deep brain stimulation.^47^ At the microcircuit level, we speculate that θ‐tACS–induced activation of cerebellar granule cells may have enhanced the inhibitory drive of Purkinje cells on the deep cerebellar nuclei,^25^, ^26^, ^27^ leading to the observed motor effects in patients with CD. Our correlation analysis emphasizes the pathophysiological implication of driving cerebellar θ oscillations in CD; a more severe CD phenotype (TWSTRS scores) corresponds to a greater detrimental effect of θ‐tACS. Patients with more severe CD may exhibit a higher susceptibility of the cerebellocortical network to increased pathological θ oscillations, potentially accounting for the greater motor impairment observed during neck‐torsion movements.

**FIG. 4:**
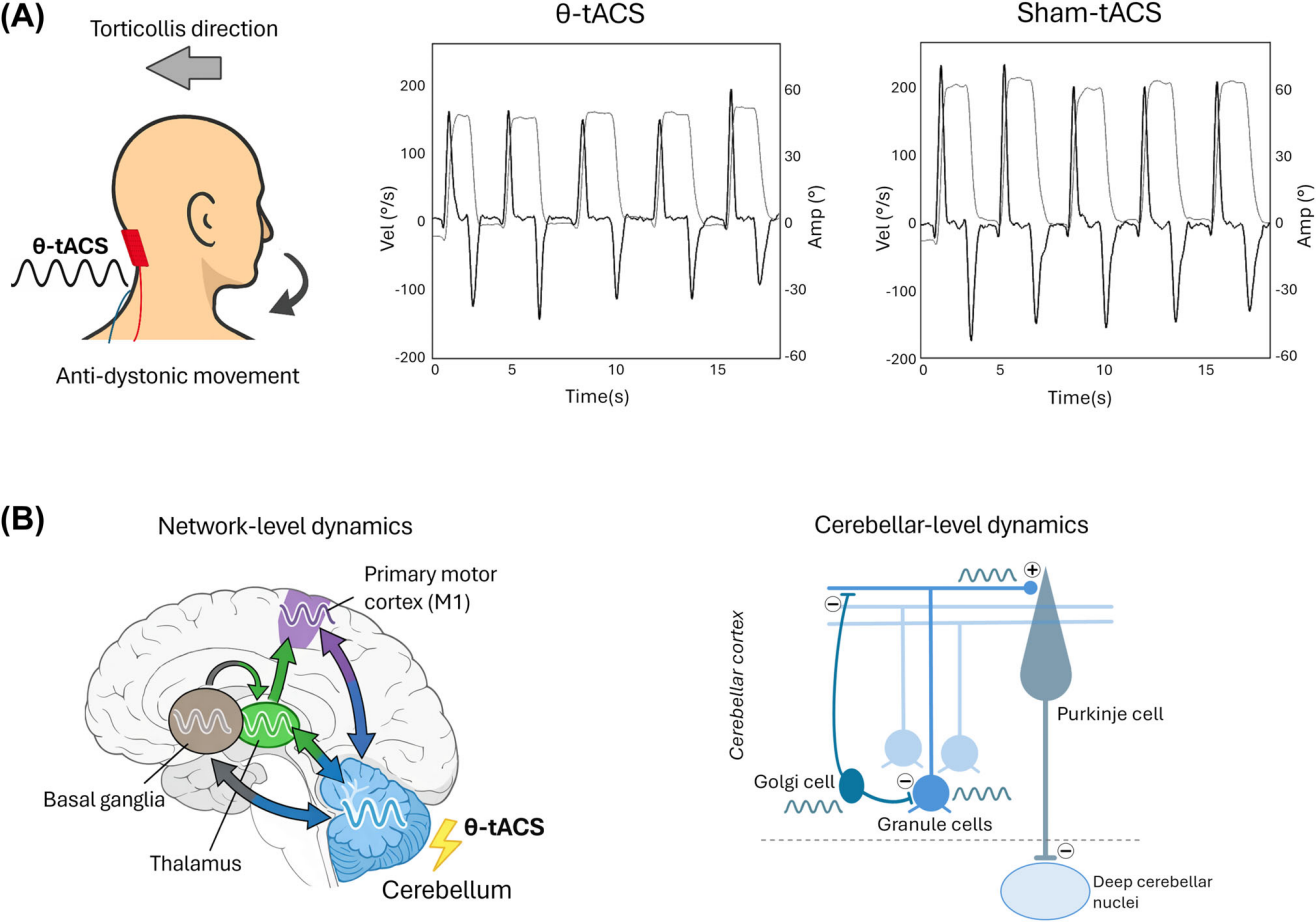
Neurophysiological effects and proposed mechanisms of cerebellar θ‐tACS (theta transcranial alternating current stimulation). (**A**) θ‐tACS applied over the cerebellar hemisphere ipsilateral to the torticollis direction induced a reduction in velocity (Vel) and amplitude (Amp) of antidystonic neck movements, as illustrated by representative kinematic traces during θ‐tACS (left plot) compared with sham‐tACS (right plot). This effect was selective for θ stimulation (not observed with β‐ or γ‐tACS), specific to antidystonic movements, and was not modified by BoNT treatment. (**B**) Proposed mechanisms of cerebellar θ‐tACS effects. Left panel: cerebello‐thalamo‐cortical and basal ganglia circuits highlighting amplification of pathological θ oscillations within the dystonia network. Right panel: simplified cerebellar microcircuit illustrating how θ‐tACS may entrain intrinsic θ oscillations of granule and Golgi cells, modulating Purkinje cell output onto deep cerebellar nuclei. [Color figure can be viewed at wileyonlinelibrary.com]

Interestingly, we observed an effect of θ‐tACS on neck torsion movements only when the stimulation was applied to the cerebellar hemisphere ipsilateral to the side of the torticollis. In contrast, no significant effects emerged when considering the stimulated hemisphere regardless of the torticollis direction. Although the laterality of cerebellar dysfunction in CD remains debated,^34^ our findings are in line with functional magnetic resonance imaging evidence showing increased activity in the cerebellar hemisphere ipsilateral to the torticollis side during head movements in the pathological direction.^48^ This suggests that ipsilateral cerebellar stimulation may preferentially influence cerebello‐thalamo‐cortical circuits, potentially by enhancing inhibitory projections to M1. Theta Golgi cells' oscillations inhibit granule cells, creating also broad lateral inhibition, which results in dense activity clusters of granule cells encoding spatiotemporal information features.^42^ Enhanced θ activity in the cerebellar cortex ipsilateral to dystonic movement may thus induce a dysregulated loop circuit, worsening neck movement in the antidystonic direction. Moreover, θ‐tACS effect was specific to antidystonic movements, possibly underlying a greater vulnerability of more impaired movements to external modulation. Prodystonic movements, which retain kinematic features closer to HCs, may rely on more preserved motor pathways and thus remain unaffected by stimulation.

Finally, although cerebellar neuronal populations are known to exhibit oscillatory activity not only in the θ band but also in β and γ frequency ranges,^28^, ^30^, ^49^ our results showed no effect of β‐ and γ‐tACS on neck movements. This may suggest that β and γ oscillations are less directly involved in the pathophysiology of CD‐related motor impairments. Alternatively, β and γ oscillations might not be critically engaged during the specific motor tasks we tested.

### Cerebellar θ‐tACS Effects Were Not Influenced by Concurrent BoNT Therapy

This finding may be explained by several factors. First, BoNT acts peripherally by weakening overactive muscles, without directly modulating cerebellar or central oscillatory activity. Second, BoNT is unlikely to modulate θ oscillations within the dystonia network, which means that θ‐tACS can amplify these rhythms irrespective of the patients' treatment state. Third, the pathophysiological role of excessive θ activity in the dystonia network may be so critical that even improvements in peripheral muscle function induced by BoNT do not counteract the central mechanism driving abnormal movements. θ oscillations may represent a novel therapeutic target in dystonia, complementing BoNT, because reducing excessive intrinsic θ activity might provide additional suppression of pathological motor output beyond what BoNT alone achieves.

### Confounds, Strengths, and Limitations of the Study

Our study employed a sham‐controlled, double‐blind design, as both patients and researchers responsible for data collection and analysis were blinded to stimulation conditions. Furthermore, the order of the different stimulation conditions was randomized and counterbalanced across participants. Patients reported very mild cutaneous sensations during stimulation, and comparable across conditions, thus making it impossible to distinguish between real and sham‐tACS. Visual sensations were limited to β‐tACS, and they were so mild that they did not interfere with the task, as confirmed by the similar performance during β‐ and sham‐tACS. To minimize possible confounds of pharmacological therapy on movement assessment, participants discontinued any oral treatments for dystonia at least 72 hours before each session. Again, sufficient intertrial intervals, equivalent to at least twice the stimulation period, were included to prevent fatigue and potential carryover effects across experimental conditions. Furthermore, the trial‐by‐trial performance was reliable in the various conditions analyzed in the study, minimizing the likelihood that observed effects were confounded by variability in movement execution.

The study's strengths include its randomized, double‐blind design, which minimizes bias, and the use of kinematic analysis, allowing for precise and objective assessment of movements. However, there are also limitations to consider. First, the relatively small sample size and restriction to patients with a predominantly torticollis phenotype may limit the generalizability of the findings. Nevertheless, this is currently the largest study investigating the effect of tACS in patients with CD. Moreover, although we attribute the observed motor detrimental effects to θ‐tACS–mediated modulation of cerebellar granule cell activity, this remains a hypothetical mechanism in the absence of direct neurophysiological evidence. Given that our study relied exclusively on kinematic outcomes, we cannot directly demonstrate modulation of cerebellar oscillatory activity or cerebellocortical interactions. Future studies should incorporate electrophysiological techniques (eg, transcranial magnetic stimulation) or neuroimaging approaches to assess changes in cerebellocortical connectivity during tACS in CD. Finally, HCs were included only to provide normative kinematic values; therefore, active tACS conditions were not tested in this group, limiting conclusions on the disease specificity of the observed effects.

### Conclusions

This study demonstrates that cerebellar θ‐tACS disrupts fast voluntary neck movements in CD, and this effect is not influenced by BoNT therapy. The selective impairment of antidystonic movements with ipsilateral stimulation indicates a direction‐specific interaction between cerebellar modulation and dysfunctional motor circuits. These findings integrate two key aspects of dystonia pathophysiology: its characterization as a network disorder involving the cerebellum and the contribution of elevated θ oscillations.^9^, ^14^, ^18^, ^19^, ^20^, ^46^ We hypothesize that pathological θ oscillatory activity may also be present at the cerebellar level and could contribute to the pathogenesis of motor symptoms in CD. Importantly, these results highlight the translational potential of targeting identified pathological oscillatory mechanisms to develop interventions aimed at restoring normal motor control in CD.

## Author Roles

(1) Research project: A. Conception, B. Organization, C. Execution; (2) Statistical analysis: A. Design, B. Execution, C. Review and critique; (3) Manuscript: A. Writing of the first draft, B. Review and critique.

D.C.: 1B, 1C, 2A, 2B, 3A

A.G.: 1A, 1B, 2A, 2B, 3B

D.B.: 1B, 1C

M.P.: 1B, 1C, 3B

M.D.R: 1C

A.S.G.: 1C

V.D.: 3B

L.A.: 1C

G.P.: 1B, 2C, 3B

M.B.: 1A, 1B, 2C, 3B

## Financial Disclosures and Conflicts of Interest

D.C. has received compensation for consultancy and speaker‐related activities from Bial. A.G. has received compensation for consultancy and speaker‐related activities from Bial, Zambon, STADA, and Medtronic. M.B. has received compensation for consultancy and speaker‐related activities from Bial and AbbVie. Author disclosures are available in the Supporting Information.

## Supporting information


**Table S1.** Additional kinematic data and statistics for the neck rotational movements in patients with cervical dystonia before therapy with botulinum toxin type A.
**Table S2.** Additional kinematic data for the neck rotational movements in patients with cervical dystonia after therapy with botulinum toxin type A (BoNT) and statistics related to the effects of cerebellar transcranial alternating current stimulation (tACS) delivered over the cerebellar hemisphere ipsilateral to the torticollis side (main effect) and BoNT treatment.

## Data Availability

The data that support the findings of this study are available from the corresponding author upon reasonable request.
