# Supplementary material for: Driving Cerebellar Theta Oscillations Interferes With Voluntary Neck Movements in Cervical Dystonia
Source: Mov Disord. 2026 Jun 10;41(10):2688–98. doi: 10.1002/mds.70394 (PMC13686109; doi:10.1002/mds.70394)
Supplement: Supplementary file 1 [file MDS-41-2688-s001.docx]

**SUPPLEMENTARY MATERIAL AND METHODS**

1. **Inclusion/exclusion criteria**

All patients primarily presented with a torticollis phenotype, with or without additional components. Those with segmental or generalized dystonia, dystonia-plus syndromes, secondary/iatrogenic forms, heredodegenerative disease, or severe neck pain were excluded. None had contraindications to tACS (1). The Montreal Cognitive Assessment (MoCA)(2) was used to exclude any cognitive impairments. Patients were asked to discontinue any pharmacological therapy for CD at least 72 hours before each experimental session.

2. **Sample size estimation**

Sample size was estimated a priori using G*Power (v3.1), based on a within-subject design including four stimulation frequencies and two movement directions. Assuming a medium effect size (f = 0.25), alpha = 0.05, and power = 0.80, a minimum of 18 participants was required to detect a difference in the effect of the factor ‘frequency’, the main aim of the study, in patients.

3. **tACS**

The impedance was kept at <10 kΩ. We used a peak-to-peak amplitude of 1.5 mA, 3-s ramp-up and down periods. Sham-tACS consisted only of ramp-up and ramp-down periods plus 1 second of stimulation at 5 Hz.

4. **Kinematic recordings**

We used an optoelectronic system (SMART motion system, BTS Engineering) with three infrared cameras to track six lightweight reflective markers placed on the trunk (two on the acromia, one on the sternum) and head (two on the frontal orbital processes, one on the nose). Participants were asked to perform rotational neck movements “as fast and wide as possible.” Each movement followed a verbal go-signal, but participants were free to initiate the movement at their own pace. Head movements were performed at 5–10 s intervals to allow return to the primary position. To ensure consistent starting positions, the head was repositioned before each go-signal.

5. **Experimental design.**

A two-minute rest followed each trial to prevent fatigue. The order of stimulation conditions was randomized and balanced across participants. Based on previous studies, tACS was turned on ~7 seconds before data acquisition and switched off immediately after (3,4), consistent with evidence showing rapid entrainment of ongoing oscillatory activity within seconds of stimulation onset (5). Both participants and recording investigators were blinded to stimulation conditions; only the investigator setting up tACS knew the parameters and did not participate in data collection or analysis, ensuring a double-blind design. Finally, although a recent study showed no long-term effects after cerebellar tACS (6), we decided to wait more than twice the stimulation duration after each kinematic trial to avoid any potential unexpected carryover effects. At the end of the first trial of each stimulation condition (pre-BoNT session), patients were asked to rate the intensity and discomfort of any visual or cutaneous sensations during stimulation on a 0–10 Visual Analog Scale, where 0 indicated ‘no sensation’ and 10 indicated ‘unbearable/intense sensation’. The questionnaire was administered by the investigator. If present, the patients were then asked whether the sensation had interfered with the task to any extent.

**Supplementary Table 1.** Additional kinematic data and statistics for the neck rotational movements in patients with cervical dystonia before therapy with botulinum toxin type-A.

|  |  | **Raw data** |  |  |  | **rmANOVA** |
| --- | --- | --- | --- | --- | --- | --- |
|  |  | **Ipsi-** |  | **Contra-** |  |  |
|  |  | **Pro-dystonic** | **Anti-dystonic** | **Pro-dystonic** | **Anti-dystonic** |  |
| **CV vel** | **Sham** | 0.117 ±  0.064 | 0.122 ±  0.052 | 0.122 ±  0.042 | 0.118 ±  0.056 | STIM FREQ: *F_3,51_*: 0.39, *P=*0.76 |
|  | **θ** | 0.150 ±  0.089 | 0.112 ±  0.063 | 0.125 ±  0.129 | 0.119 ±  0.062 | STIM SIDE: *F_1,17_*: 0.08, *P=*0.78 |
|  | **β** | 0.127 ± 0.065 | 0.107 ±  0.053 | 0.109 ± 0.050 | 0.152 ± 0.129 | MOV DIR: *F_1,17_*: 0.21, *P=*0.65 |
|  | **γ** | 0.125 ± 0.067 | 0.122 ±  0.040 | 0.113 ± 0.069 | 0.104 ± 0.069 | STIM FREQ x STIM SIDE: *F_3,51_*: 0.71, *P=*0.55  STIM FREQ x MOV DIR: *F_3,51_*: 0.95, *P=*0.42  STIM SIDE x MOV DIR: *F_1,17_*: 2.77, *P=*0.11  STIM FREQ x STIM SIDE x MOV DIR: *F_3,51_*: 0.89, *P=*0.45 |
| **CV amp** | **Sham** | 0.065 ± 0.098 | 0.112 ± 0.203 | 0.035 ±  0.040 | 0.060 ± 0.099 | STIM FREQ: *F_3,51_*: 1.43, *P=*0.24 |
|  | **θ** | 0.075 ±  0.160 | 0.068 ± 0.144 | 0.034 ±  0.027 | 0.083 ±  0.117 | STIM SIDE: *F_1,17_*: 1.08, *P=*0.31 |
|  | **β** | 0.041 ± 0.033 | 0.091 ± 0.150 | 0.039 ± 0.033 | 0.114 ± 0.199 | MOV DIR: *F_1,17_*: 2.33, *P=*0.14 |
| **CV mov** | **γ**  **Sham**  **θ**  **β**  **γ** | 0.098 ± 0.175  0.079 ± 0.056  0.084 ±  0.060  0.077 ± 0.047  0.083 ± 0.066 | 0.115 ± 0.190  0.098 ± 0.070  0.109 ± 0.107  0.101 ± 0.077  0.140 ± 0.147 | 0.043 ± 0.043  0.077 ± 0.047  0.127 ± 0.129  0.071 ± 0.043  0.083 ± 0.037 | 0.130 ± 0.235  0.091 ± 0.078  0.117 ± 0.134  0.090 ± 0.075  0.093 ± 0.077 | STIM FREQ x STIM SIDE: *F_3,51_*: 1.00, *P=*0.40  STIM FREQ x MOV DIR: *F_3,51_*: 0.72, *P=*0.54  STIM SIDE x MOV DIR: *F_1,17_*: 0.95, *P=*0.34  STIM FREQ x STIM SIDE x MOV DIR: *F_3,51_*: 0.61, *P=*0.61  STIM FREQ: *F_3,51_*: 0.50, *P=*0.69  STIM SIDE: *F_1,17_*: 0.06, *P=*0.81  MOV DIR: *F_1,17_*: 3.69, *P=*0.07  STIM FREQ x STIM SIDE: *F_3,51_*: 1.00, *P=*0.40  STIM FREQ x MOV DIR: *F_3,51_*: 1.38, *P=*0.26  STIM SIDE x MOV DIR: *F_1,17_*: 1.38, *P=*0.26  STIM FREQ x STIM SIDE x MOV DIR: *F_3,51_*: 1.56, *P=*0.21 |

CV vel: coefficient of variation of peak angular velocity: CV amp: coefficient of variation of movement amplitude; CV mov: coefficient of variation of the mean inter-movement interval; Ipsi-: ipsilateral cerebellar hemisphere to the side of dystonia; Contra-: contralateral cerebellar hemisphere to the side of dystonia; Sham: sham-tACS; ϑ: theta-tACS; β: beta-tACS; γ: gamma-tACS; rmANOVA: repeated-measures analysis of variance; STIM FREQ: factor ‘stimulation frequency; STIM SIDE: factor ‘stimulation side’; MOV DIR: factor ‘movement direction’. Data reflect mean values ± 1 standard deviation.

**Supplementary Table 2.** Additional kinematic data for the neck rotational movements in patients with cervical dystonia after therapy with botulinum toxin type-A (BoNT) and statistics related to the effects of cerebellar tACS delivered over the cerebellar hemisphere ipsilateral to the torticollis side (main effect) and BoNT treatment.

|  |  | **Raw data** |  |  |  | **rmANOVA** |
| --- | --- | --- | --- | --- | --- | --- |
|  |  | **Ipsi-** |  | **Contra-** |  |  |
|  |  | **Pro-dystonic** | **Anti-dystonic** | **Pro-dystonic** | **Anti-dystonic** |  |
| **CV vel** | **Sham** | 0.097 ±  0.053 | 0.117 ±  0.057 | 0.106 ±  0.059 | 0.115 ±  0.053 | STIM FREQ: *F_3,45_*: 1.79, *P=*0.16 |
|  | **θ** | 0.121 ±  0.066 | 0.123 ±  0.055 | 0.132 ±  0.095 | 0.099 ±  0.038 | SESS: *F_1,15_*: 0.33, *P=*0.57 |
|  | **β** | 0.123 ± 0.072 | 0.150 ±  0.055 | 0.110 ± 0.065 | 0.124 ± 0.065 | MOV DIR: *F_1,15_* 0.35, *P=*0.56 |
|  | **γ** | 0.132 ± 0.065 | 0.128 ±  0.039 | 0.086 ± 0.057 | 0.096 ± 0.045 | STIM FREQ x MOV DIR: *F_3,45_*: 0.91, *P=*0.44  STIM FREQ x SESS: *F_3,45_*: 1.31, *P=*0.28  SESS x MOV DIR: *F_1,15_*: 0.59, *P=*0.45  STIM FREQ x SESS x MOV DIR: *F_3,45_*: 0.84, *P=*0.48 |
| **CV amp** | **Sham** | 0.031 ± 0.025 | 0.077 ± 0.135 | 0.081 ±  0.131 | 0.083 ± 0.108 | STIM FREQ: *F_3,45_*: 0.25, *P=*0.86 |
|  | **θ** | 0.072 ±  0.117 | 0.118 ± 0.142 | 0.065 ±  0.097 | 0.141 ±  0.249 | SESS: *F_1,15_*: 0.98, *P=*0.34 |
|  | **β** | 0.044 ± 0.067 | 0.110 ± 0.237 | 0.028 ± 0.024 | 0.081 ± 0.141 | MOV DIR: *F_1,15_* 1.69, *P=*0.21 |
| **CV mov** | **γ**  **Sham**  **θ**  **β**  **γ** | 0.033 ± 0.028  0.074 ± 0.086  0.078 ±  0.053  0.065 ± 0.036  0.084 ± 0.053 | 0.052 ± 0.056  0.082 ± 0.040  0.090 ± 0.062  0.090 ± 0.048  0.066 ± 0.047 | 0.102 ± 0.156  0.099 ± 0.069  0.655 ± 2.240  0.113 ± 0.104  0.093 ± 0.082 | 0.075 ± 0.101  0.070 ± 0.038  0.104 ± 0.072  0.096 ± 0.089  0.092 ± 0.046 | STIM FREQ x MOV DIR: *F_3,45_*: 0.64, *P=*0.59  STIM FREQ x SESS: *F_3,45_*: 2.44, *P=*0.08  SESS x MOV DIR: *F_1,15_*: 0.11, *P=*0.75  STIM FREQ x SESS x MOV DIR: *F_3,45_*: 0.21, *P=*0.89  STIM FREQ: *F_3,45_*: 0.42, *P*=0.74  SESS: *F_1,15_*: 3.70, *P*=0.07  MOV DIR: *F_1,15_*: 10.36, ***P*=0.06**  STIM FREQ x MOV DIR: *F_3,45_*: 0.17, *P*=0.92  STIM FREQ x SESS: *F_3,45_*: 0.58, *P*=0.63  SESS x MOV DIR: *F_1,15_*: 2.532, *P*=0.15  STIM FREQ x SESS x MOV DIR: *F_3,45_*: 2.01, *P*=0.13 |

CV vel: coefficient of variation of peak angular velocity: CV amp: coefficient of variation of movement amplitude; CV mov: coefficient of variation of the mean inter-movement interval; Ipsi-: ipsilateral cerebellar hemisphere to the side of dystonia; Contra-: contralateral cerebellar hemisphere to the side of dystonia; Sham: sham-tACS; ϑ: theta-tACS; β: beta-tACS; γ: gamma-tACS; rmANOVA: repeated-measures analysis of variance; STIM FREQ: factor ‘stimulation frequency; SESS: factor ‘session’; MOV DIR: factor ‘movement direction’. Data reflect mean values ± 1 standard deviation.

**SUPPLEMENTARY REFERENCES**

1.	Antal A, Alekseichuk I, Bikson M, Brockmöller J, Brunoni AR, Chen R, et al. Low intensity transcranial electric stimulation: Safety, ethical, legal regulatory and application guidelines. Clin Neurophysiol. 1 settembre 2017;128(9):1774–809. doi:10.1016/j.clinph.2017.06.001

2.	Nasreddine ZS, Phillips NA, Bédirian V, Charbonneau S, Whitehead V, Collin I, et al. The Montreal Cognitive Assessment, MoCA: a brief screening tool for mild cognitive impairment. J Am Geriatr Soc. aprile 2005;53(4):695–9. doi:10.1111/j.1532-5415.2005.53221.x PubMed PMID: 15817019.

3.	Fabbrini A, Guerra A, Giangrosso M, Manzo N, Leodori G, Pasqualetti P, et al. Transcranial alternating current stimulation modulates cortical processing of somatosensory information in a frequency- and time-specific manner. NeuroImage. 1 luglio 2022;254:119119. doi:10.1016/j.neuroimage.2022.119119 PubMed PMID: 35321858.

4.	Guerra A, Colella D, Giangrosso M, Cannavacciuolo A, Paparella G, Fabbrini G, et al. Driving motor cortex oscillations modulates bradykinesia in Parkinson’s disease. Brain. 1 gennaio 2022;145(1):224–36. doi:10.1093/brain/awab257

5.	Johnson L, Alekseichuk I, Krieg J, Doyle A, Yu Y, Vitek J, et al. Dose-dependent effects of transcranial alternating current stimulation on spike timing in awake nonhuman primates. Sci Adv. 2 settembre 2020;6(36):eaaz2747. doi:10.1126/sciadv.aaz2747

6.	Spampinato D, Avci E, Rothwell J, Rocchi L. Frequency-dependent modulation of cerebellar excitability during the application of non-invasive alternating current stimulation. Brain Stimul Basic Transl Clin Res Neuromodulation. 1 marzo 2021;14(2):277–83. doi:10.1016/j.brs.2021.01.007 PubMed PMID: 33482375.
